# Changes in Olsen Phosphorus Concentration and Its Response to Phosphorus Balance in Black Soils under Different Long-Term Fertilization Patterns

**DOI:** 10.1371/journal.pone.0131713

**Published:** 2015-07-15

**Authors:** Xiaoying Zhan, Li Zhang, Baoku Zhou, Ping Zhu, Shuxiang Zhang, Minggang Xu

**Affiliations:** 1 Institute of Agricultural Resources and Regional Planning, Chinese Academy of Agricultural Sciences/ National Engineering Laboratory for Improving Quality of Arable Land, Beijing, 100081, P. R. China; 2 Soil and Fertilizer Institute, Heilongjiang Academy of Agricultural Sciences, Harbin, Heilongjiang 150086, P. R. China; 3 Center of Agricultural Environment and Resources, Jilin Academy of Agricultural Sciences, Gongzhuling, Jilin 136100, P. R. China; Chinese Academy of Sciences, CHINA

## Abstract

The Olsen phosphorus (P) concentration of a soil is a key index that can be used to evaluate the P supply capacity of the soil and to estimate the optimal P fertilization rate. A study of the relationship between the soil Olsen P concentration and the P balance (P input minus P output) and their variations among different fertilization patterns will help to provide useful information for proper management of P fertilization. In this paper, the two investigated long-term experiments were established on black soils in the northeast region of China. Six fertilization treatments were selected: (1) unfertilized (CK); (2) nitrogen only (N); (3) nitrogen and potassium (NK); (4) nitrogen and phosphorus (NP); (5) nitrogen, phosphorus, and potassium (NPK); and (6) nitrogen, phosphorus, potassium and manure (NPKM). The results showed that the average Olsen P concentrations in the black soils at Gongzhuling and Harbin (16- and 31-year study periods, respectively), decreased by 0.49 and 0.56 mg kg^-1^ a^-1^, respectively, without P addition and increased by 3.17 and 1.78 mg kg^-1^ a^-1^, respectively, with P fertilization. The changes in soil Olsen P concentrations were significantly (*P*<0.05) correlated with the P balances at both sites except for the NP and NPK treatments at Gongzhuling. Under an average deficit of 100 kg ha^-1^ P, the soil Olsen P concentration at both sites decreased by 1.36~3.35 mg kg^-1^ in the treatments without P addition and increased by 4.80~16.04 mg kg^-1 ^in the treatments with 100 kg ha^-1^ of P accumulation. In addition, the changes in Olsen P concentrations in the soil with 100 kg ha^-1^of P balance were significantly correlated with the P activation coefficient (PAC, percentage of Olsen P to total P, r=0.99, *P*<0.01) and soil organic matter content (r=0.91, *P*<0.01). A low pH was related to large changes of Olsen P by 1 kg ha^-1^ of P balance. These results indicated that soil organic matter and pH have important effects on the change in soil Olsen P by 1 kg ha^-1 ^of P balance.

## Introduction

Soil phosphorus (P) deficiencies can result in the imbalance of soil nutrient and low crop yields in agricultural practice. In this case, P fertilizer must be applied to the soils to improve the soil P levels and crop production. However, about 80%-90% of the added inorganic P fertilizer becomes unavailable to crops in the year of application due to adsorption and precipitation with Ca, Fe and Al in soils [1, 2]. These mineral associated P in the soil can be converted among them, and the conversion processes could have effect on the available P level [3]. Available P in the soil, which is the most effective P sink, has been considered an important indicator for evaluating the capacity of the soil to supply P and for estimating the P fertilization rate and P loss risk from runoff [4, 5]. Soil Olsen P is a routine available P index of soil in north China. Therefore, agronomic P management strategies can be based on the changes in soil Olsen P [6].

The changes in soil Olsen P following the application of P fertilizer (P input) have been examined in many studies [7–9]. Along with the high P uptake of crops, changes in soil Olsen P are driven by the P balance (P input minus P output) actually [10–12]. Moreover, a significantly positive linear correlation existed between the change in soil Olsen P concentration and the P balance that was first considered by the Rothamsted Experimental Station [13]. Related studies in the UK, India and China showed that each 100 kg P ha^-1^ P surplus increase the Olsen P by 1~6 mg P kg^-1^ [10, 14, 15]. The variations among the seven long-term experimental sites located in different provinces across China were attributed to the different environments, crop systems and soil physico-chemical properties, as well as suitable temperatures and higher clay content that enhanced the change in Olsen P by each 100 kg ha^-1^ P accumulation [16].

At a particular site that has the same environmental and crop system conditions, the soil physico-chemical properties caused by various fertilizations are the sole factors that have an impact on the relationship between the Olsen P and P balance of the treatments [17, 18]. The change in Olsen P by each 1 kg ha^-1^ P balance indicates the P activating ability from sparingly P to available P. Therefore, the soil properties that impact sparingly P activation (desorption, dissolution and mineralization) should be considered. It has been proven that various fertilization patterns can increase or decrease the soil pH and organic matter content [19, 20]. The soil pH is one of the most important factors affecting P activation process, including sorption-adsorption, precipitation-solubilization and other chemical reactions related to P fractions transformation [21]. For example, study found that when the rhizosphere pH of rape seedlings decreased from 6.5 to 4, the rhizosphere available P increased by 10 times after the growth of 35 days [22]. Studies have also proved that the pH reduction mainly increases the dissolution of Ca associated P [23]. Soil organic matter is another factor that activates the sparingly P. Because the soil organic matter can decrease the P adsorption due to the competing adsorption sites by organic anion; and dissolve the mineral associated P by low-molecular-weight organic acids [24]. For black loess soils in Gansu, China, the Olsen P concentration decreased by 3.18 mg kg^-1^ (control) and 1.95 mg kg^-1^ (nitrogen fertilizer only) per 100 kg ha^-1^ of P deficit and increased by 0.29~3.85 mg kg^-1^ per 100 kg ha^-1^ of P accumulation when P (chemical P and manure) was added [17]. Few papers have focused on the variations and especially their possible mechanism(s) of different soil physico-chemical properties among different fertilization patterns. Black soil, which is a typical soil in Northeast China, plays an important role in Chinese crop production. In Northeast China, understanding the variations and their possible mechanism(s) that result in the changes in Olsen P and the P balance under different fertilization patterns is useful for predicting the Olsen P dynamics and optimal P fertilization of black soils.

The hypothesis of this study is that soil pH and organic matter would be important influence factors that affect the differences in changes in Olsen P by 1 kg ha^-1^ P balance among fertilization patterns. Here, we developed a minimal dataset of the changes in the Olsen P and P balance of two long-term experimental sites located on the black soil. The objectives of this study were to (i) investigate the temporal changes in the Olsen P concentration under long term fertilization; (ii) quantify the relationship between the Olsen P concentration and the P balance under different treatments; and (iii) determine the effects of soil pH and organic matter on the changes in Olsen P by 1 kg ha^-1^ P balance under long-term fertilization in Northeast China.

## Materials and Methods

### Experimental sites

The two long-term experimental sites, which were established in 1989 and 1979, are located in Gongzhuling Jilin province and Harbin Heilongjiang province, respectively, in Northeast China. The authorities of the two field sites are the Jilin Academy of Agricultural Sciences and the Heilongjiang Academy of Agricultural Sciences of China, both of which granted permission for the research work. No specific field permits were required for this study, and no locations used in our study involved endangered or protected species. The study period presented in this paper is from 1989 to 2005 at Gongzhuling (16 years) and from 1979 to 2010 at Harbin (31 years). The black soils at the two sites are classified as Udic Mollisols [25]. The climatic types of the two sites are mid-temperate, semi-humid, continental monsoon climate. Descriptions of the experimental sites and the initial soil (0–20 cm) properties are summarized in Table 1.

**Table 1:** Site characteristics and initial soil (0–20 cm) properties of two long-term experimental sites.

Site	Gongzhuling	Harbin
Location	43°30′N, 124°48′E, 220 m altitude	45°40′N, 126°35′E, 151 m altitude
Evaporation (mm)	1400	1425
Precipitation (mm)	550	533
Mean annual temp. (°C)	4.5	3.5
Cropping system	Continuous maize	Wheat-soybean-maize rotation
Clay content (<0.002 mm, %)	32.1	12.9
Organic matter (g kg^-1^)	22.8	26.7
Total N (g N kg^-1^)	1.40	1.47
Total P (g P kg^-1^)	0.61	1.07
Total K (g K kg^-1^)	18.42	20.88
Alkaline-hydrolyzable N(mg N kg^-1^)	114	151
Olsen P (mg P kg^-1^)	11.79	22.27
NH_4_OAc-extractable K(mg K kg^-1^)	158	200
pH(2.5 water/soil)	7.60	7.22

### Experimental design

The Gongzhuling and Harbin experimental plots covered 400 and 168 m^2^, respectively. Numerous treatments were implemented at these two sites, and six treatments that included no P, chemical P, and chemical and organic P were selected at each site: unfertilized (CK); nitrogen only (N); nitrogen and potassium (NK); nitrogen and phosphorus (NP); nitrogen, phosphorus, and potassium (NPK); and nitrogen, phosphorus, potassium and manure (NPKM) (Table 2). These two long term experiments were not replicated. Urea, diammonium phosphate or superphosphate, and potassium chloride (KCl) or potassium sulfate (K_2_SO_4_) were used as the N, P, and K fertilizers, respectively, at the two sites. The organic fertilizer was pig manure (30 t ha^-1^, equivalent to 115 kg ha^-1^ N and 52 kg ha^-1^ P) at Gongzhuling and horse dung (18.6 t ha^-1^, equivalent to 75 kg ha^-1^ N and 25 kg ha^-1^ P) at Harbin [26]. The inorganic and organic fertilizers were applied annually at Gongzhuling before seeding. At Harbin, chemical fertilizers were applied each fall after harvest, and organic fertilizer was only applied after maize culture [27].

**Table 2:** Rates of annual N, P and K applied as chemical fertilizer (kg ha^-1^ a^-1^).

Site	Crop name	N	NK	NP	NPK	NPKM
Gongzhuling	Maize	165-0-0*	165-0-68.5	165-36-0	165-36-68.5	50-36-68.5
Harbin	Wheat	150-0-0	150-0-62.2	150-32.75-0	150-32.75-62.2	150-32.75-62.2
	Maize	150-0-0	150-0-62.2	150-32.75-0	150-32.75-62.2	150-32.75-62.2
	Soybean	75-0-0	75-0-62.2	75-65.5-0	75-65.5-62.2	75-65.5-62.2

* “165-0-0” represents the amount of applied chemical fertilizers, N, P and K, in order.

### Soil and plant sampling and chemical analysis

Soil samples were collected from the top 20 cm of the soil, which was considered the active depth, after crop harvest each year. Five fresh soil samples were randomly sampled from the middle area of each plot. The soil samples were mixed, air-dried and sieved through 2.0 mm mesh screens for the determination of available nutrients, and through 0.25 mm mesh screens prior to total nutrient analyses. Subsamples were stored prior to analyzing their physicochemical properties. Total soil P was digested with H_2_SO_4_-HClO_4_ and measured using the molybdenum-blue colorimetric method. The soil Olsen P concentration was determined using 0.5 mol L^-1^ NaHCO_3_ (pH 8.5) and the molybdenum-blue colorimetric method. In addition, other initial soil samples were analyzed for organic matter, total nitrogen, total potassium, alkaline-hydrolyzable N, NH_4_OAc-extractable K and pH. The grains and straws of wheat and maize were manually harvested, dried at 70°C to a uniform moisture level, weighed and then the P concentrations were measured separately. All the indexes mentioned above were analyzed according to the methods presented by Lu [28].

### Calculations and statistical analysis

The P activation coefficient (PAC, %) represents the proportion of Olsen P to total P [29]. The PAC (%) was calculated as follows:

$$PAC=\frac{Po}{Pt}\times 100\;,$$

where Po is the concentration of Olsen P (mg kg^-1^), and Pt is the concentration of total P (mg kg^-1^) in the soil.

The change in soil Olsen P (ΔOlsen P, mg kg^-1^) was calculated as follows:

ΔOlsenP=Pi−P0 ,

where Pi is the soil Olsen P concentration (mg kg^-1^) at year i and P0 is the initial concentration of soil Olsen P(mg kg^-1^).

The crop P offtake (P_C_, kg ha^-1^ a^-1^) was calculated as follows:

$$P_{C}=Y_{G}\times C_{G}+Y_{S}\times C_{S\;,}$$

where Y_G_ is the grain yield(kg ha^-1^), C_G_ is the grain P concentration (%), Y_S_ is the straw yield (kg ha^-1^) and C_S_ is the straw P concentration (%).

The annual apparent P balance in the soil was calculated as the P input minus the P output each year. The P balance was calculated as the sum of the annual apparent P balance, which represents the total cumulative P in the surface layers (0–20 cm) of the soil. This study was based on the soil surface (0–20 cm) balance but not the potential losses resulting from runoff and soil erosion [30]. Thus, the P balance (kg ha^-1^) was calculated as follows:

$$Pbalance=\sum \left(P_{F}-P_{C}\right)\;,$$

where P_F_ is the P application (kg ha^-1^ a^-1^).

The average level of soil organic matter and soil PAC was analyzed using one-way ANOVA in SAS V8 (SAS Institute, USA). The means were compared using the Duncan method. The significance level used in this paper is *P* = 0.05. A linear regression was used to determine the relationships between the ΔOlsen P and the P balance, the Olsen P changes by each 100 kg ha^-1^ P balance and the PAC, the Olsen P changes by each 100 kg ha^-1^ P balance and the organic matter, and the changing trends of the Olsen P in soil under various fertilization patterns.

## Results

### The Olsen P concentrations in black soils

**Fig 1:**
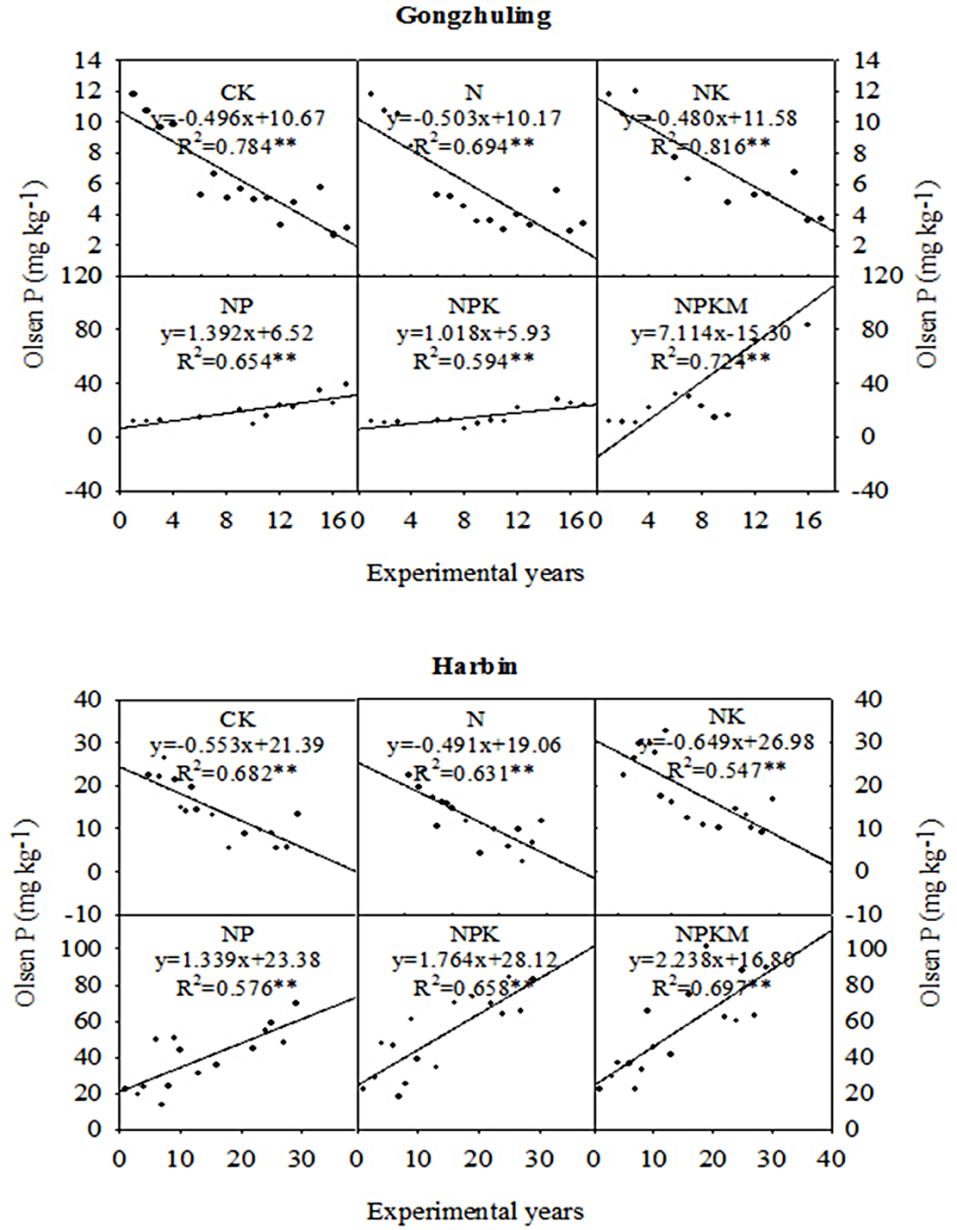
The Olsen P concentrations in the black soils under long-term fertilization.

The number of experimental years that were selected for this study was 16 years for Gongzhuling and 31 years for Harbin. Without the application of P fertilizer (CK, N and NK), the Olsen P concentration declined significantly (*P*<0.01) over time at the two sites. The Olsen P concentration decreased by 0.48~0.50 mg kg^-1^ a^-1^ at Gongzhuling and by 0.49~0.65 mg kg^-1^ a^-1^ at Harbin. But we cannot compare the Olsen P decrease at the two sites due to the difference of fertilization years. So, what is the situation of Olsen P decrease after 16-year at Harbin? Data showed that the Olsen P concentration of CK, N and NK decreased at a rate of approximately 0.50, 0.50 and 0.48 mg kg^-1^ a^-1^ at Gongzhuling, and at 1.17, 1.10 and 1.14 mg kg^-1^ a^-1^ at Harbin after the same 16 years’ experiment. Generally, the Olsen P concentration increased over time at the two sites, and the correlation coefficients were significant (*P*<0.01) for this relationship. The order of the increase in the Olsen P concentration at the Gongzhuling site was NPKM>NP>NPK. After 16 years of fertilization at the Gongzhuling site, the NPK treatment had an Olsen P concentration that was 184% greater than that of the NK treatment, and the NPKM treatment showed an increase of 217% relative to the NPK treatment. The order of the increase in the Olsen P concentration at the Harbin site was NPKM>NPK>NP. After 31 years of fertilization at this site, the NPK treatment yielded an Olsen P concentration that was 249% greater than that of the NK treatment, while the addition of manure improved the soil Olsen P concentration by 8% (Fig 1).

### P balance in black soils

**Fig 2:**
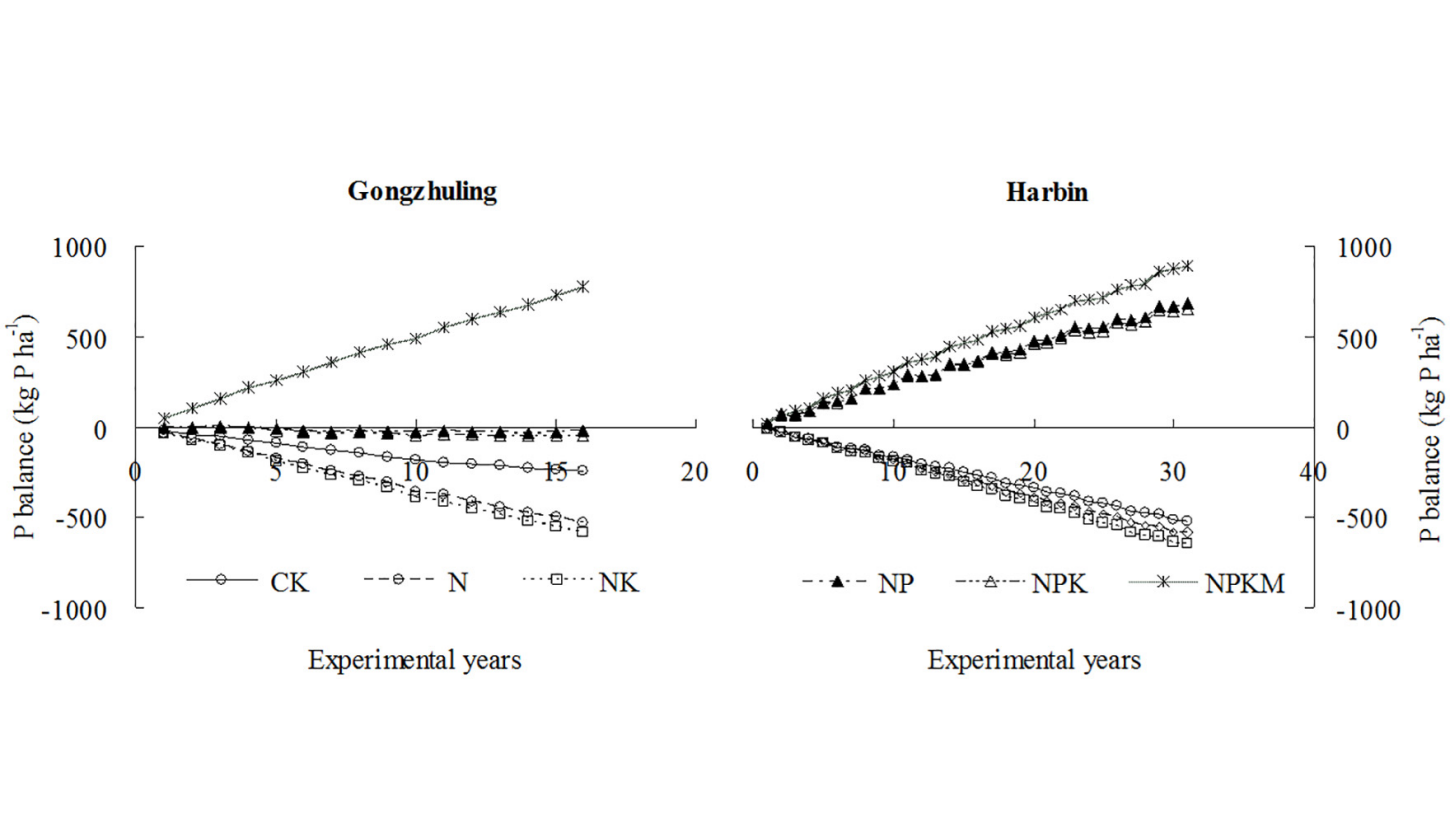
The P balances of the black soils under long-term fertilization at Gongzhuling and Harbin.

The soil P content was depleted each year in the three no-P treatments at both sites. After the 16- and 31-year fertilization periods, the P contents of the Gongzhuling and Harbin sites were depleted by 245 and 518 kg ha^-1^ in the CK, 527 and 586 kg ha^-1^ in the N, and 585 and 648 kg ha^-1^ in the NK treatments, respectively. The P balances were -20.22, -47.20, and 778 kg ha^-1^ for the NP, NPK and NPKM treatments, respectively, after 16 years of P fertilization at Gongzhuling. At Harbin, 687, 656 and 894 kg ha^-1^ of P was accumulated after 31 years in the NP, NPK and NPKM treatments, respectively (Fig 2).

### The response of the change in the Olsen P concentrations to the P balance of the black soils

**Fig 3:**
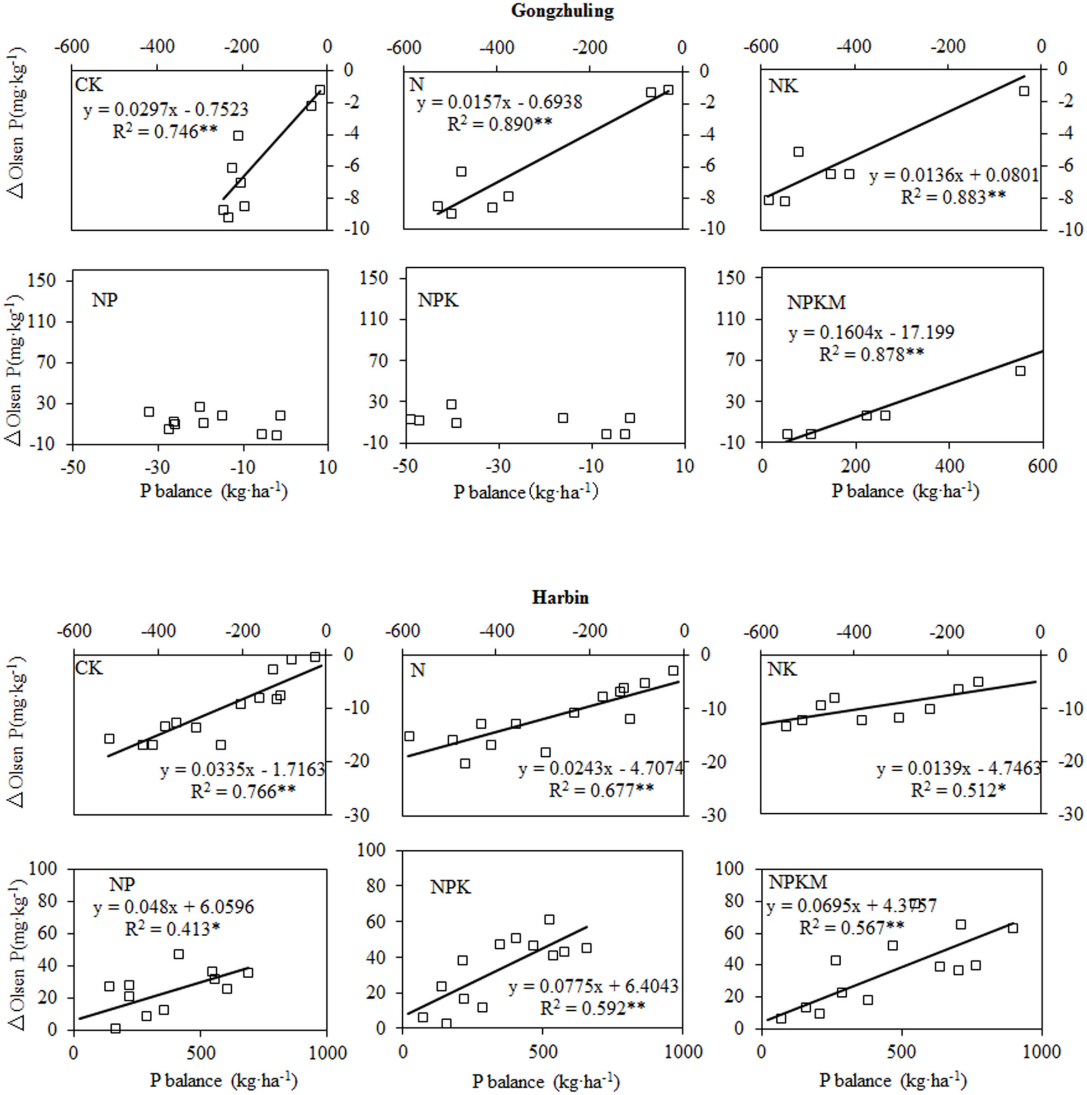
Relationships between the changes in the soil Olsen P and the P balances of the black soils.

The responses of the changes in the Olsen P concentrations to the P balances of the two black soils are shown in Fig 3. In the regression equation, x indicates the P balance of the soil, and y indicates the change in the soil Olsen P (ΔOlsen P) concentration. Thus, the slope of the regression equation represents a change in the Olsen P (mg kg^-1^) concentration per unit of P surplus or deficit (kg ha^-1^ P) [31].

A significant positive linear relationship was observed between the changes in the soil Olsen P concentration and P balance for all fertilization patterns except the NP and NPK treatments at Gongzhuling. The soil Olsen P concentration decreased by 2.97, 1.57 and 1.36 mg kg^-1^ for for each 100 kg ha^-1^ P deficit in the CK, N and NK treatments, respectively, at Gongzhuling. At Harbin, the Olsen P decreased by more than that observed at Gongzhuling, with values of 3.35, 2.43 and 1.39 mg kg^-1^ for the CK, N and NK treatments, respectively. In addition, the changes in the Olsen P concentrations for each 100 kg ha^-1^ P deficit were larger at Harbin than Gongzhuling.

For each 100 kg ha^-1^ P surplus, the Olsen P concentration increased by 16.04 mg kg^-1^ under the NPKM treatment at Gongzhuling. The Olsen P concentration increased at the Harbin site in the following order: NPK>NPKM>NP (i.e. 7.75, 6.95, 4.80 mg kg^-1^, respectively), and the NPKM value was considerably higher, for Gongzhuling than for Harbin. In addition, the increases in the Olsen P concentrations with each 1 kg ha^-1^ P for the P treatments were greater than the decreases in the treatments without P addition.

### Change of soil pH and organic matter in black soils

**Fig 4:**
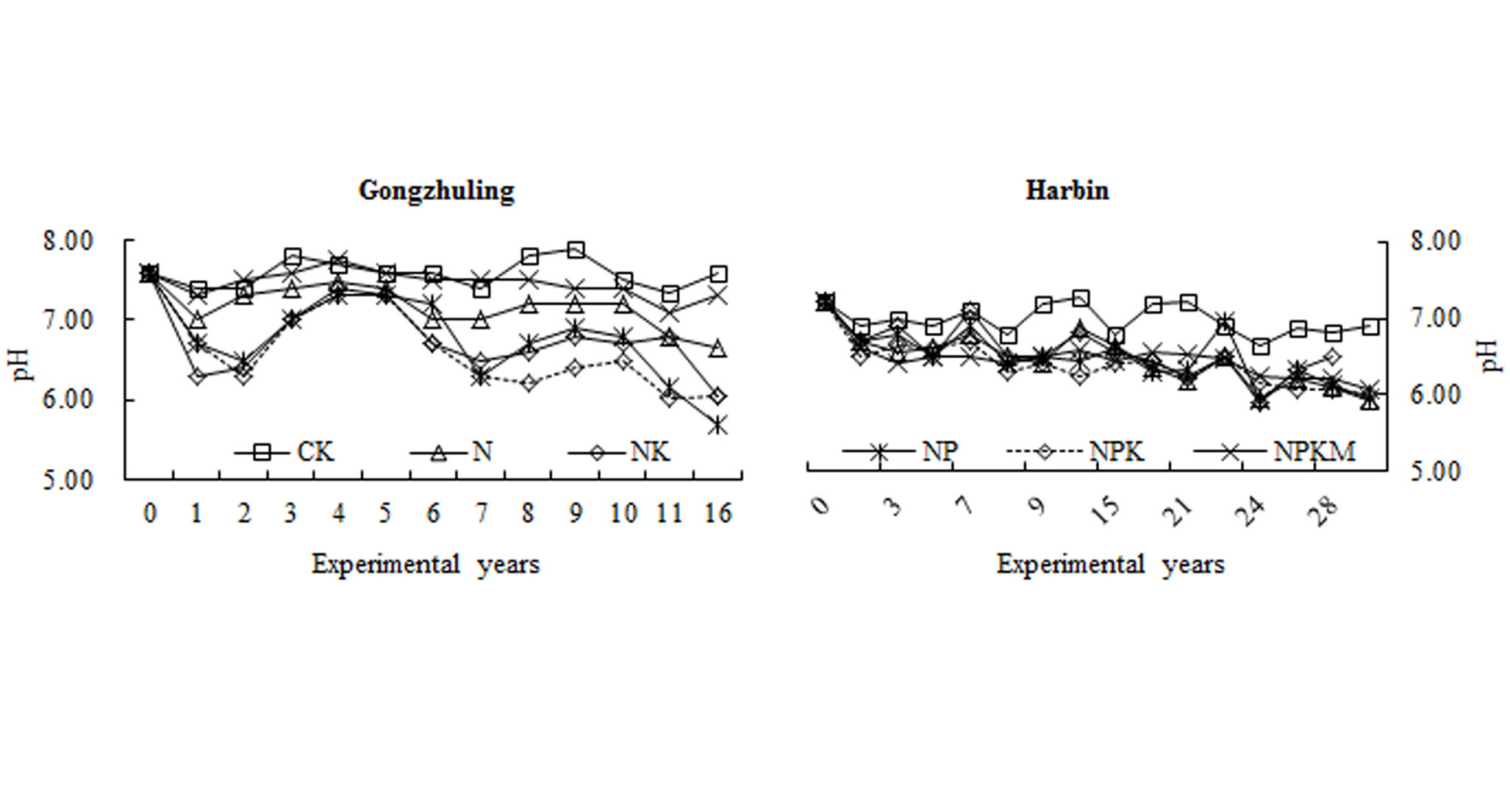
Changes in the pH of the black soils under long-term fertilization at Gongzhuling and Harbin.

Soil pH remained stable under the CK treatment, and declined under all fertilizer application treatments with experimental years due to N fertilization at the two sites. At Gongzhuling, the decrease value of pH was about 1.5 units for chemical fertilizer treatments and 0.3 units for NPKM after 16-year fertilization; at Harbin, the pH value declined about 1.2 units for all fertilizer treatments after 31-year fertilization. That means that chemical fertilizer plus manure (NPKM) slowed down the decrease of pH in comparison of NPK at Gongzhuling, but not for Harbin, which is due to the different amount and type of manure (Material and method) (Fig 4).

**Fig 5:**
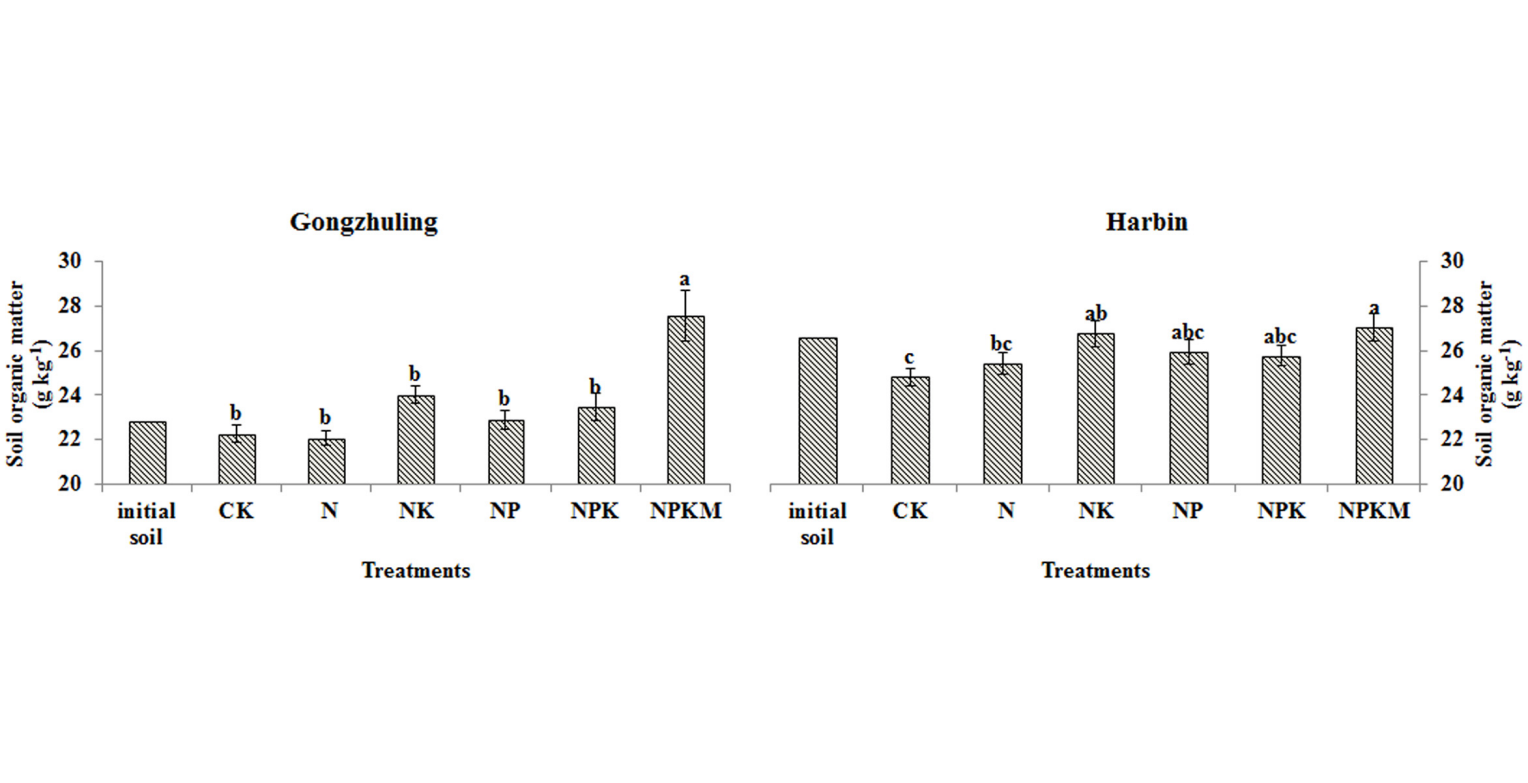
Average level of soil organic matter under long-term fertilization at Gongzhuling and Harbin. Note: Values are significantly different (*P*<0.05) between fertilization treatments (a, b, c) if followed by different letters.

Fig 5 shows the average level of soil organic matter after long term fertilizer at the two sites. After 16-year experiment, soil organic matter of NPKM was significant higher than other five treatments, and the NPKM significantly enhanced the soil organic matter level by 17.3% in comparison with NPK at Gongzhuling. For the site of Harbin, the lowest organic matter level was the CK treatment and the highest level was the NPKM treatment. There was no significant difference between NPK and NPKM.

## Discussion

### The Olsen P concentrations changed with year, fertilization and the P balance

Without P fertilizer (CK, N and NK), the Olsen P concentration significantly (*P*<0.01) decreased at the two sites. Soil Olsen P concentration at Harbin decreased faster in comparison with Gongzhuling after the same 16 years’ experiment. Colomb [32] suggests that in the absence of fertilizer, the conversation rate of soil available P from inorganic and organic P depends on the initial P level. Therefore, the more rapid decrease at Harbin in this study could be related to its higher initial Olsen P concentration (22.27 mg kg^-1^) compared with Gongzhuling (11.79 mg kg^-1^). This viewpoint was also expressed by Qu [8, 9] who studied 11 typical Chinese soils in a long-term experiment and found that soils with higher initial Olsen P concentrations resulted in more rapid decreases in Olsen P. When P fertilizer was applied, the Olsen P concentration increased especially when chemical and manure fertilizers were used together. Similar results have been reported in other papers [33].

Negative P balances were observed in all treatments that did not receive any P fertilizer input (Fig 2), and the decreases in the soil Olsen P concentrations were significantly (*P*<0.05) correlated with the P balances at both sites (Fig 3). A decrease in the Olsen P decrease by each 100 kg ha^-1^ P balance was observed at Harbin (CK, N and NK) and Gongzhuling (CK, N and NK); a greater decrease occurred at Harbin for each of these treatments. This result likely occurred because of the greater initial Olsen P concentration at Harbin. In addition, the Harbin soil had lower clay content (12.9%), which presented a low soil P sorption capacity [34, 35], compared with the Gongzhuling (32.1%) soil. However, Cao [16] proved that the soil clay content was significantly positively correlated with the change in of Olsen P across six long term experimental sites in China. This may be attributed to a decrease in phosphate run off as a result of higher clay content [36].

Generally, positive P balances are observed in most P application treatments [3, 12], and the Olsen P concentration increases under a P surplus [11]. In this study, the Olsen P concentrations increased significantly as a result of a P surplus, except for the NP and NPK treatments at Gongzhuling (Fig 1). This result occurred because the P balances (Fig 2) of the NP and NPK treatments were nearly 0, and the Olsen P concentration also increased with the number of experimental years (Fig 1). In addition, the soil total P of these two treatments, which verified the result of the P balance, changed only a little (NP: an increase of 5% and NPK: a decrease of 11%) in comparison with the other four treatments at the 16-year experiment. This result potentially occurred because when there was no P surplus, various forms of residual soil P, e.g., Ca-P, Fe-P, Al-P could interchange and transferred to Olsen P through a dynamic P transformation [33]. These dynamic processes include the transformation of the inorganic P fractions to available P through desorption and dissolution, and the organic P fractions to the available P fraction by mineralization [1, 37]. The inorganic and organic P fractions should to be analyzed to explain the difference in the Olsen P change by each 100 kg ha^-1^ P balance.

### Relationships between the changes in the soil Olsen P by each 100 kg ha^-1^ P balance and the P activation coefficient

Within a site, the change in the soil Olsen P caused by each 100 kg ha^-1^ P balance for the different fertilizer patterns was affected by the soil properties, including the soil P activation coefficient (PAC), soil organic matter and pH [18, 38].

**Fig 6:**
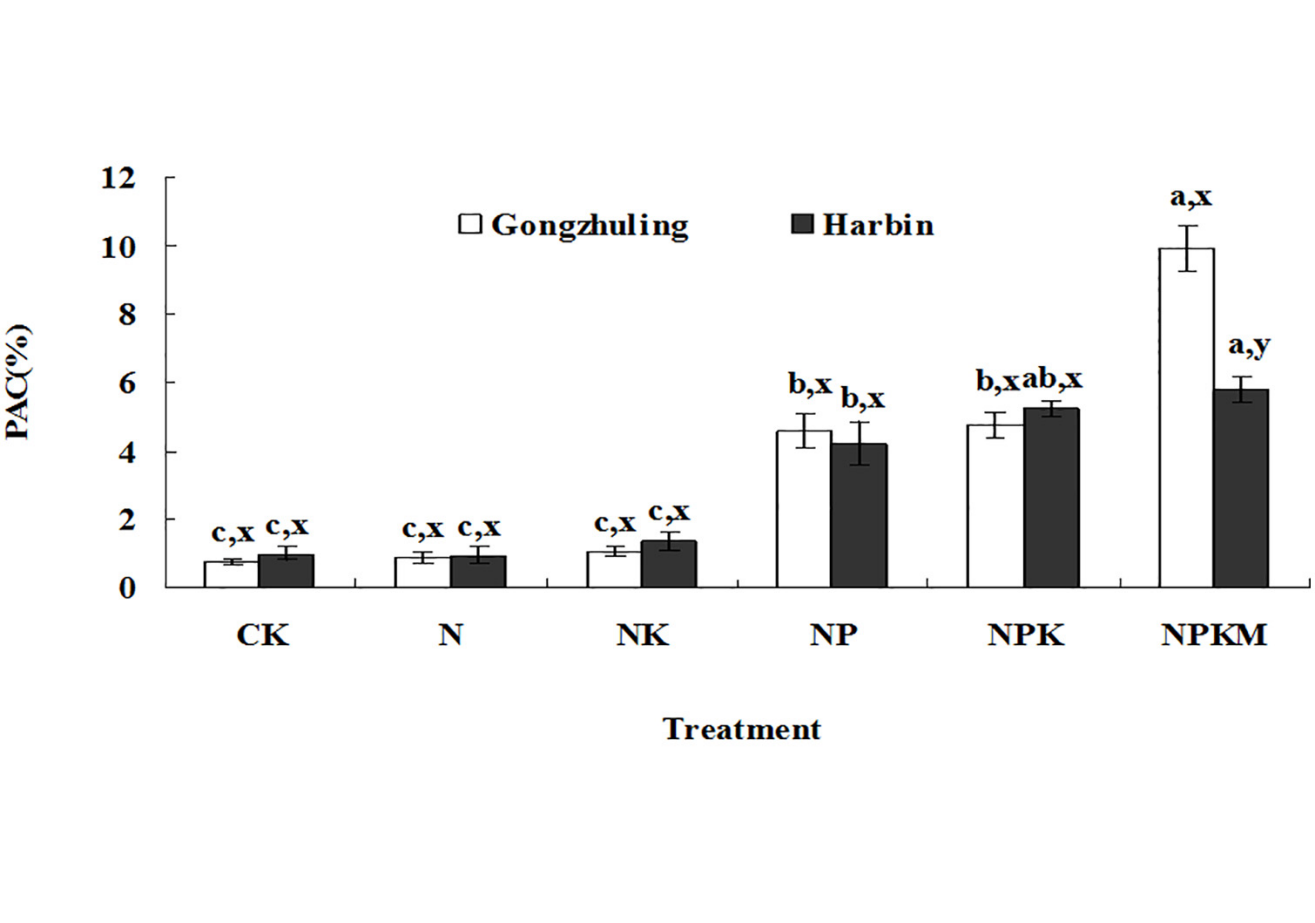
Soil PAC in black soils under long-term fertilization. Note: Values are significantly different (*P*<0.05) between fertilization treatments (a, b, c) and between sites in the same treatment (x, y) if followed by different letters.

**Fig 7:**
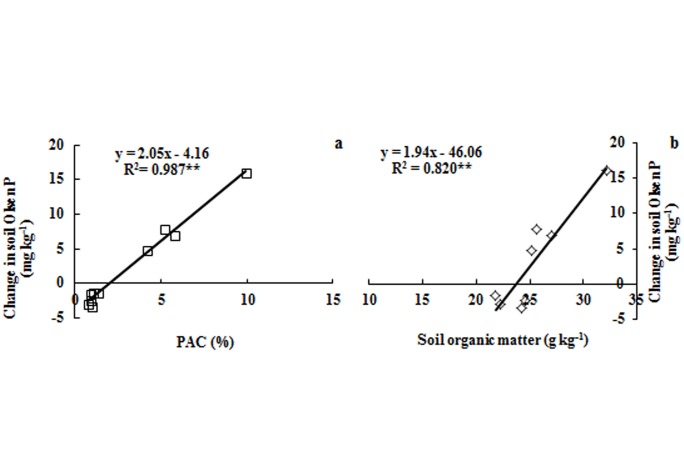
Relationships between the of PAC, soil organic matter and changes in the soil Olsen P in black soils under long-term fertilization. Note: To distinguish the no P and P addition treatments, the changes in soil Olsen P by each 100 kg ha^-1^ P balance of no P treatments (CK, N and NK) were defined to the negative values in this figure.

The proportion of the soil Olsen P relative to the total P (PAC) indicates the capacity of the soil to bind P in relatively soluble fractions [18]. Greater PAC values indicate that the soil total phosphorus is more easily converted to Olsen P. The PAC value of the P treatments was much greater than that of the no P treatments (Fig 6). This could verify that the increase in Olsen P caused by each 100 kg ha^-1^ P surplus was larger than the decrease in Olsen P caused by each 100 kg ha^-1^ P depletion. The reason is that the P application can result in a greater P desorption ability compared with those treatments where P is not applied [39, 40]. Selles [41] also found that the increase in Olsen P by each 100 kg ha^-1^ P surplus was substantially greater than the decrease in Olsen P by each 100 kg ha^-1^ P depletion in no P treatments in two wheat production systems in a 39-year study in Canada. Under the combined application of organic manure and chemical fertilizer in this study, the soil PAC was 5.8% at Harbin and 9.9% at Gongzhuling, and these values differed significantly. This result is similar to the observed changes in the soil Olsen P by each 100 kg ha^-1^ P balance between the two sites. The statistical data (Fig 7a) showed that the changes in the soil Olsen P by each 100 kg ha^-1^ P balance were significantly correlated with the PAC (r = 0.99, *P*<0.01).

### Relationships between changes in soil Olsen P by each 100 kg ha^-1^ P balance, soil organic matter and pH

The different fertilization patterns had obvious effects on soil organic matter level [42], and this viewpoint is similar with us (Fig 5). The organic matter play an important role on P availability by decreasing the P adsorption due to the competing adsorption sites by organic anion and dissolving the mineral associated P by low-molecular-weight organic acids [24]. In this study, the statistical data (Fig 7b) showed that changes in the soil Olsen P by each 100 kg ha^-1^ P balance were strongly related to the soil organic matter (r = 0.91, *P*<0.01) across all treatments at the two sites. Shen [43] found that the change in the soil Olsen P by each 100 kg ha^-1^ P balance in the NPK treatment was lower than that of the NPKM treatment at three long term experimental sites (Changping, Yangling and Zhengzhou) in China. However, in this paper, compared with NPK, the NPKM treatment significantly increased the Olsen P (Fig 1) and resulted in P accumulation at Harbin (Fig 2), but did not affect the value of the changes in the soil Olsen P by each 100 kg ha^-1^ P balance (Fig 3), Pei [17] obtained the same result on black loess soil. In addition, the change in the soil Olsen P by each 100 kg ha^-1^ P balance for the chemical fertilizer plus manure at Gongzhuling was much greater than Harbin (Fig 3) and Pingliang [17]. It can be concluded that chemical fertilizer plus manure does not always enhance the changes in the soil Olsen P by each 100 kg ha^-1^ P balance compared with NPK, and the situation depends on the P availability of the manure. In this paper, the organic P of horse dung (Harbin) is difficult to activate, and pig manure (Gongzhuling) contains highly active organic P according the study [44]. That is to say, if the organic P in manure mineralizes readily, the soil Olsen P will increase rapidly. Future research should focus on the organic P activity of manure.

During the long term experiment, soil pH was often influenced by fertilizations. The pH values for N and NK treatments reduced in comparison with CK at the two sites (Fig 4), as urea behaves similar to ammonia and H^+^ ions are produced during the ammonication-nitrication process [45]. Lower pH value can promote more dissolution of sparingly P into Olsen P [23]. This could explain why the change in soil Olsen P by each 100 kg ha^-1^ P balance decreased less in the N and NK treatments than in the CK treatment. For the treatment of NPKM, the humus acid of manure can prevent soil from acidifying due to its buffering action [24]. So the average pH value in the NPK treatment (6.16) during the previous 9 years was lower than that in NPKM (6.24) treatment. More P activation at lower pH may be one of the reasons that NPK expressed higher changes in the soil Olsen P concentration by each 100 kg ha^-1^ P balance than NPKM at Harbin site. Meanwhile, Zhang [46] showed that the acid phosphatase contents that were related to soil pH in the N and NK treatments were higher than those measured in the CK treatment. This can potentially explain the smaller decrease in the soil Olsen P concentration changes by each 100 kg ha^-1^ P balance in the N and NK treatments relative to the CK treatment. Acid phosphatase can slow the decrease in Olsen P because acid phosphatase is an important root exudate that hydrolyzes organic P [47].

## Conclusion

1. In this study, average Olsen P concentrations in the black soils at Gongzhuling (16-year) and Harbin (31-year) decreased by 0.49 and 0.56 mg kg^-1^ a^-1^ without P addition and increased by 3.17 and 1.78 mg kg^-1^ a^-1^ with P fertilization.
2. Under an average deficit of 100 kg ha^-1^ P, the soil Olsen P concentration at both sites decreased by 1.36~3.35 mg kg^-1^ in the treatments without P addition and increased by 4.80~16.04 mg kg^-1^ in the P treatments with 100 kg ha^-1^ of P accumulation.
3. In addition, the soil with the higher initial Olsen P content and lower clay content resulted in more rapid decreases in Olsen P when P was not added; the P fertilization models with higher soil organic matter lower pH resulted in more rapid increase rates of Olsen P by 100 kg ha^-1^ P balance.
